# Effects of caffeinated and decaffeinated coffee on biological risk factors for type 2 diabetes: a randomized controlled trial

**DOI:** 10.1186/1475-2891-10-93

**Published:** 2011-09-13

**Authors:** Nicole M Wedick, Aoife M Brennan, Qi Sun, Frank B Hu, Christos S Mantzoros, Rob M van Dam

**Affiliations:** 1 Department of Nutrition, Harvard School of Public Health, Boston, MA, USA; 2 Division of Endocrinology, Diabetes & Metabolism, Beth Israel Deaconess Medical Center, Harvard Medical School, Boston, MA, USA; 3 Department of Epidemiology, Harvard School of Public Health, Boston, MA, USA; 4 Channing Laboratory, Department of Medicine, Brigham and Women's Hospital, and Harvard Medical School, Boston, MA, USA; 5 Section of Endocrinology, Boston VA Healthcare System, Harvard Medical School, Boston, MA, USA; 6 Department of Environmental Health, Harvard School of Public Health, Boston, MA, USA

## Abstract

**Background:**

Coffee consumption has been associated with a lower risk of type 2 diabetes in prospective cohort studies, but the underlying mechanisms remain unclear. The aim of this study was to evaluate the effects of regular and decaffeinated coffee on biological risk factors for type 2 diabetes.

**Methods:**

Randomized parallel-arm intervention conducted in 45 healthy overweight volunteers who were nonsmokers and regular coffee consumers. Participants were assigned to consumption of 5 cups (177 mL each) per day of instant caffeinated coffee, decaffeinated coffee, or no coffee (i.e., water) for 8 weeks.

**Results:**

Average age was 40 years and body mass index was 29.5 kg/m^2^. Compared with consuming no coffee, consumption of caffeinated coffee increased adiponectin (difference in change from baseline 1.4 μg/mL; 95% CI: 0.2, 2.7) and interleukin-6 (difference: 60%; 95% CI: 8, 138) concentrations and consumption of decaffeinated coffee decreased fetuin-A concentrations (difference: -20%; 95% CI: -35, -1). For measures of glucose tolerance, insulin sensitivity, and insulin secretion, no significant differences were found between treatment groups.

**Conclusions:**

Although no changes in glycemia and/or insulin sensitivity were observed after 8 weeks of coffee consumption, improvements in adipocyte and liver function as indicated by changes in adiponectin and fetuin-A concentrations may contribute to beneficial metabolic effects of long-term coffee consumption.

**Trial Registration:**

clinicaltrials.gov NCT00305097

## Introduction

Coffee consumption has been associated with a substantially lower risk of type 2 diabetes mellitus (T2DM) in prospective cohort studies in the United States (US), Europe, and Asia [1,2]. Paradoxically, caffeine intake acutely reduced insulin sensitivity and glucose tolerance in short-term trials [3]. Data on the effects of coffee intake on glucose metabolism from randomized trials lasting more than 24 hours are sparse and limited to effects on fasting glucose and insulin concentrations [4]. In addition to caffeine, coffee contains hundreds of components and decaffeinated coffee, chlorogenic acid, quinides, lignans, and trigonelline significantly improved insulin sensitivity or glucose levels in animal studies [5-7].

In cross-sectional studies, coffee consumption has been associated with higher adiponectin concentrations, lower concentrations of inflammatory markers, and lower levels of markers of liver damage [8,9]. In a recent non-randomized trial, increases in coffee consumption were also associated with increases in adiponectin levels [10]. Higher adiponectin levels have been consistently associated with a lower risk of T2DM possibly reflecting anti-inflammatory and insulin sensitizing effects [11]. Fetuin-A, a biomarker for inflammation and liver function, is a glycoprotein secreted by the hepatocytes with effects on insulin signaling via inhibition of the insulin receptor tyrosine kinase in both liver and skeletal tissue [12]. Higher fetuin-A levels have been associated with insulin resistance and a higher risk of T2DM [13,14]. Inflammatory markers including interleukin-6 (IL-6) have also been implicated in the development of insulin resistance [15].

To our knowledge, the effects of regular and decaffeinated coffee consumption on adiponectin, fetuin-A, and inflammatory markers have not been studied in randomized controlled trials. We therefore conducted an 8-week parallel-arm randomized trial to determine the effects of caffeinated and decaffeinated coffee on risk factors for T2DM.

## Research design and methods

### Study Participants

The study was conducted between May 2006 and September 2008. Participants were volunteers recruited from the community and were deemed eligible to participate if they were regular coffee consumers (≥ 2 cups/day), nonsmokers, aged 18 years or older, and overweight (body mass index (BMI) 25-35 kg/m^2^), but otherwise healthy according to a detailed screening that included a medical history, physical examination, electrocardiogram, and laboratory tests. Participants were excluded if they had diabetes mellitus (fasting blood glucose ≥ 7 mmol/L), heart disease, stroke, hypertension (blood pressure > 140 mmHg/90 mmHg or antihypertensive medications), alcoholism, drug abuse, abnormal hepatic function (transaminases > twice the upper limit of normal), abnormal renal function (creatinine > 97.2 μmol/L), malabsorption syndromes, gastro-esophageal reflux disease or a history of ulcer. We also excluded individuals on medications with potential interactions with caffeine or that might impact study results, and women who were breastfeeding or planning a pregnancy.

Of the 65 individuals that participated in the screening, 11 did not qualify to participate and 9 withdrew consent prior to randomization. Of the remaining 45 individuals, three participants discontinued between the baseline and 4-week visits, and one participant dropped out after the 4-week visit (See Additional file 1, **figure s1**). The study was approved by the institutional review boards of the Beth Israel Deaconess Medical Center and the Harvard School of Public Health and all participants provided written informed consent to participate. The clinical trial registration number is NCT00305097.

### Study Design

Participants were counseled by a research dietitian prior to starting the study and asked to abstain from coffee and caffeine-containing foods for the duration of the study (except for the coffee provided as part of the treatment regimen) and received a list of caffeine containing foods and beverages to avoid. After two weeks of caffeine washout and after an overnight fast (minimum 12 hours), participants attended the clinical research center for baseline assessment. Physical examination and baseline blood draw were performed followed by an oral glucose tolerance test (OGTT).

At this visit, participants were randomly assigned by a blinded statistician using a randomization schedule generated via the PROC PLAN procedure in the Statistical Analysis System 9.1 (SAS Institute Inc., NC, US). The treatment allocation was 1:1:1 (caffeinated coffee: decaffeinated coffee: no coffee) in block sizes of six with stratification by sex. A surrogate randomization list was generated and validated by the randomization statistician. Participants, investigators, and laboratory staff were blinded regarding the treatment assignment for the caffeinated and decaffeinated coffee arms.

Participants were asked to maintain stable exercise and dietary habits throughout the study and were asked to keep 3-day food diaries (1 weekend day and 2 weekdays) prior to each visit. Participants returned for repeat assessments after four weeks and 8 weeks. Participants were assessed for changes in medications or health status, for side-effects of coffee and for adherence to the intervention by questionnaires. Participants had an additional non-fasting visit at 6 weeks (typically scheduled between 12-2 pm) for a blood draw to measure serum caffeine concentrations for compliance assessment.

### Intervention

Participants who were assigned to one of the two coffee arms of the study were provided with five pre-weighed two-gram portions of instant coffee per day (caffeinated or decaffeinated Nestlé's Taster's Choice^®^). They were instructed to prepare the coffee with 6 ounces (177 mL) of boiling water and to consume coffee five times per day with every meal, and at mid-morning and mid-afternoon. Participants who reported using sugar in their coffee were provided with a non-caloric sweetener and participants had the choice of using a non-dairy creamer, which was also provided. Participants who were randomized to the control (no coffee) intervention were instructed to consume five 6-ounce glasses of water also with every meal, and at mid-morning and mid-afternoon.

Caffeine content of the coffee was measured at Tufts University School of Medicine (Boston, MA, US). Chlorogenic acid was measured at the RIKILT Institute of Food Safety (Wageningen, The Netherlands) as previously described [16]. Trigonelline content was measured in quadruplicate at the Canterbury Health Laboratories (Christchurch, New Zealand) using methods described by Lever et al. [17]. The caffeine concentration in the prepared regular coffee was 344 μg/mL. Chlorogenic acid and trigonelline were measured both in samples kept at room temperature during the study and samples that were stored in a freezer at the beginning of the study. Chlorogenic acid concentrations were 34 mg/g (frozen: 33 mg/g) for regular and 25 mg/g (frozen: 23 mg/g) for decaffeinated coffee and trigonelline concentrations were 8.7 mg/g (frozen: 8.7 mg/g) for regular and 7.4 mg/g (frozen: 7.1 mg/g) for decaffeinated coffee. Therefore, the 5 daily cups of caffeinated coffee provided 345 mg caffeine, 302 mg chlorogenic acid, and 78 mg trigonelline. Five cups of decaffeinated coffee provided 216 mg chlorogenic acid and 65 mg trigonelline.

### Measurements

At the baseline, 4-week and 8-week visits, participants wearing light clothing and no shoes had weight, height and waist circumference measured by a trained investigator. Waist circumference was measured half way between the iliac crest and the lower border of the ribcage. Body composition was measured using Tanita (model Quantum II, Lean Body software, RJL Systems, Clinton Township, MI, US) single frequency bioelectrical impedance analysis. Blood pressure was measured under fasting conditions using a validated oscillometric device with appropriately sized cuffs (Dinamap Pro100, Critikon, Tampa, FL). At each assessment, 3 blood pressure readings, taken in intervals of five minutes, were averaged.

An intravenous cannula was placed in the antecubital fossa and blood samples were collected after fasting for at least 12 hours. Subsequently, participants consumed 75 grams of glucose (7.5 ounces of glucola) and blood samples were taken at 30, 60 and 120 minutes. Glucose was measured by glucose hexokinase method and lipids using an autoanalyzer in the central clinical laboratory at the Beth Israel Deaconess Medical Center. All other samples were stored in a liquid nitrogen freezer at < -130°C until assayed in duplicate with all samples for the same participant included in the same batch. Double-antibody radioimmunoassay (Immulite^® ^Chemiluminescence, Siemens Co., New York, NY, US) was used to measure insulin [intra-assay coefficient of variation (CV) 2.1%], C-reactive protein (CRP) (CV 3.6%), and IL-6 (CV 4.7%). Adiponectin (CV 3.9%) and fetuin-A (CV 2.4%) levels were measured by enzyme linked immunosorbent assay kits (Millipore Corporation, Billerica, MA, US and Alpco, Salem, NH, US, respectively).

At the 6-week visit, a non-fasting blood sample was taken for measurement of serum caffeine levels. Plasma concentrations of caffeine and metabolites (i.e., theobromine, paraxanthine, theophylline) were determined by high-performance liquid chromatography at the Tufts University School of Medicine with within- and between-assay CVs of 9% or lower.

### Statistical Analysis

We compared the differences in change in biomarkers from baseline to the 8-week visit between the treatment groups using an analysis of covariance model. The model included the change from baseline as the dependent variable with treatment group as a main effect and baseline values of the dependent variable as an additional covariate. Weight change from baseline was also included as a covariate. Differences between treatment groups were based on linear contrasts for (1) caffeinated versus no coffee, (2) decaffeinated versus no coffee, and (3) caffeinated versus decaffeinated coffee. The adjusted mean with standard error was reported by treatment, and 95% confidence intervals (CIs) were computed. Statistical significance was evaluated at an alpha level of 0.05.

Glucose and insulin area under the curve (AUC) values during the OGTT were calculated using the trapezoidal rule [18]. The composite insulin sensitivity index (CISI) was calculated using the formula: [10,000/the square root of (fasting glucose × fasting insulin) × (mean glucose × mean insulin)] [19]. The homeostasis model assessment for insulin resistance (HOMA-IR) was calculated as [(fasting glucose × fasting insulin)/22.5] [20]. The ratio between the 30 minute increment in insulin to 30 minute increment in glucose concentration (insulinogenic index) was used as a measure of early insulin secretion. Variables with obvious departures from normality were log-transformed, then converted back to their original scale to yield geometric means and associated 95% CIs. Normality was achieved for these variables after log-transformation. For log-transformed variables, mean differences between groups yield a ratio given the principles of logged numbers and differences for log-transformed variables are therefore presented as percentages.

Statistical analysis was performed using the last-observation-carried-forward method for imputation of missing values. One observation was carried forward from the Week 4 visit, except for fetuin-A which was not measured at week four. The final Week 8 analysis was based on 41 participants as three participants discontinued after baseline and one participant had a missing value for change from baseline in weight. For glucose homeostasis measurements, data were based on 40 participants because one participant additionally had a missing value for fasting glucose. Outliers for the outcome variables were identified using a criterion of ± three times the interquartile range. We performed sensitivity analyses to test differences between the treatment groups in changes in risk factors after outliers were removed. Because results remained essentially the same as for the main analysis and statistically significant differences remained significant, we only present results without removal of outliers. Spearman correlation coefficients between adiponectin, IL-6, and fetuin-A and markers of glucose metabolism were calculated at baseline, as well as correlating changes over the 8-week intervention period. All statistical analyses were performed using SAS.

## Results

The mean age of participants was 40 years and the mean BMI 29.5 kg/m^2^. Table 1 shows the baseline characteristics of the participants according to group allocation. Except for the lower IL-6 and CRP concentrations in the caffeinated coffee group, there were no substantial differences between the groups. Correlation coefficients for adiponectin, CRP, IL-6 and fetuin-A concentrations with markers of glucose homeostasis at baseline are shown in Table 2. Forty-one participants (91%) completed the 8-week intervention period. The intervention did not result in significant differences between treatment groups in body composition, diet, physical activity, and sleep duration (Additional file 1, **table s1**). To assess compliance, caffeine concentrations were measured in non-fasting blood collected during the 6-week visit. Participants were unaware that caffeine was being measured at this visit. Caffeine concentrations were 3.1, 0.3, and 0.2 μg/mL for the caffeinated coffee, decaffeinated coffee, and no coffee group, respectively (*p*-value < 0.0001) and similar differences were found for caffeine metabolites (Additional file 1, **figure s2**).

**Table 1:** Baseline characteristics and clinical measurements for study participants by treatment group

	**Caffeinated Coffee**	**Decaffeinated Coffee**	**No Coffee**
			
	**(N = 16)**	**(N = 14)**	**(N = 15)**
	
			
Age (years)	38.7 (7.3)	41.9 (14.6)	41.2 (17.4)
Sex (% female)	11 (68.8)	9 (64.3)	9 (60.0)
Usual caffeinated coffee intake (mL/d)	998 (432)	885 (509)	758 (500)
Ethnicity (% non-Hispanic white)	10 (62.5)	10 (71.4)	10 (66.7)
Physical activity (MET-hrs/wk)	28.5 (31.6)	37.1 (35.4)	31.6 (18.0)
Weight (kg)	79.8 (10.7)	86.5 (9.3)	84.6 (11.4)
Body mass index (kg/m^2^)	29.0 (2.3)	29.5 (2.2)	30.0 (2.4)
Waist circumference (cm)			
Men	97.7 (7.3)	102.5 (7.3)	102.6 (6.3)
Women	85.2 (10.3)	90.9 (7.8)	88.3 (7.4)
Fat mass (%)^a^	32.0 (7.5)	33.5 (8.9)	34.3 (6.6)
Glucose (mmol/L)^b^			
Fasting	4.8 (0.5)	4.9 (1.0)	4.7 (0.4)
2-hour	5.8 (1.8)	5.7 (1.6)	6.2 (1.6)
Area under the curve	13.2 (2.4)	13.3 (3.6)	14.0 (2.1)
Insulin (pmol/L)			
Fasting	48.4 (33.6)	71.7 (50.9)	52.6 (21.1)
2-hour	288.7 (132.6)	381.1 (223.3)	366.4 (180.0)
Area under the curve	574. 2 (317.7)	717.8 (395.7)	675.1 (275.3)
CISI (mmol/L^-1^*pmol/L^-1^)	24.2 (15.4)	20.8 (15.7)	17.2 (7.4)
Insulin _30 min_/Glucose _30 min_	86.7 (59.2)	163.9 (162.4)	118.8 (89.6)
HOMA-IR	1.7 (1.1)	2.5 (2.0)	1.9 (0.8)
Adiponectin (μg/mL)	7.8 (4.8)	7.1 (3.7)	8.6 (3.6)
C-reactive protein (mg/L)	1.2 (1.0)	4.6 (5.8)	2.8 (3.2)
Interleukin-6 (pg/mL)	1.0 (0.5)	2.6 (2.6)	2.0 (1.3)
Fetuin-A (μg/mL)	246.1 (45.3)	270.7 (37.4)	280.2 (56.9)
			
HDL-cholesterol (mmol/L)	1.5 (0.4)	1.5 (0.5)	1.5 (0.3)
LDL-cholesterol (mmol/L)	2.8 (0.9)	2.4 (0.8)	2.3 (0.7)
Triglycerides (mmol/L)	1.1 (0.7)	1.0 (0.4)	1.0 (0.5)
			
Systolic blood pressure (mmHg)	115.0 (10.6)	119.7 (13.8)	121.3 (10.7)
Diastolic blood pressure (mmHg)	69.8 (7.6)	71.8 (5.8)	73.2 (8.7)
Heart rate (beats per minute)	68.0 (8.2)	71.3 (10.4)	66.5 (9.3)

Unless otherwise noted, data are means (standard deviation) for continuous variables and number (percentages) for categorical variables.

CISI, composite insulin sensitivity index. HOMA, homeostasis model assessment; IR, insulin resistance, HDL, high density lipoprotein; LDL, low density lipoprotein.

^a ^Assessed by bioelectric impedance analysis.

^b ^One participant missing baseline fasting glucose measurement (Decaffeinated coffee treatment group).

**Table 2:** Correlation coefficients for adiponectin, C-reactive protein, interleukin-6, and fetuin-A concentrations and markers of glucose metabolism at baseline

	Adiponectin (μg/mL)	C-reactive Protein (mg/L)^a^	Interleukin-6 (pg/mL)^a^	Fetuin-A (μg/mL)^a^
				
Glucose (mmol/L)^b^				
Fasting^a^	-0.29	0.15	0.004	0.11
2-hour	-0.25	0.16	0.20	0.26
Area under the curve	-0.22	0.13	0.05	0.02
Insulin (pmol/L)				
Fasting^a^	-0.06	0.23	0.17	0.33^c^
2-hour^a^	-0.21	0.30	0.43^c^	0.30
Area under the curve	-0.17	0.22	0.43^c^	0.31^c^
CISI (mmol/L^-1 ^* pmol/L^-1^)^a^	0.13	-0.26	-0.27	-0.33^c^
Insulin_30 min_/Glucose_30 min_^b^	-0.11	0.06	0.40^c^	0.24
HOMA-IR^b^	-0.04	0.22	0.15	0.34^c^

Spearman correlation coefficients have been adjusted for age, sex, and baseline BMI.

CISI, composite insulin sensitivity index; HOMA, homeostasis model assessment.

^a ^Values were log transformed due to significant departures from normality.

^b ^One participant missing baseline fasting glucose measurement (Decaffeinated coffee treatment group).

^c ^*P*-value < 0.05.

Table 3 shows the differences in biomarkers between treatment groups at the end of the intervention adjusted for baseline values and change in weight. Compared with consuming no coffee, consumption of caffeinated coffee and decaffeinated coffee was not significantly associated with changes in glucose AUC (caffeinated: -0.78 mmol/L; 95% CI: -2.52, 0.95; decaffeinated: 0.47 mmol/L; 95% CI: -1.40, 2.33) or 2-hour plasma glucose (caffeinated: -0.41 mmol/L; 95% CI: -1.64, 0.83; decaffeinated: 0.79 mmol/L; 95% CI: -0.58, 2.16).

**Table 3:** Risk factors for type 2 diabetes by coffee treatment group at Week 8^a^

	Adjusted means at Week 8 value (95% CI)		
	Caffeinated Coffee	Decaffeinated Coffee	No Coffee
Glucose (mmol/L)			
Fasting^†^	4.97 (4.58, 5.40)	5.07 (4.60, 5.58)	4.78 (4.37, 5.22)
2-hour	5.48 (4.66, 6.30)	6.67 (5.72, 7.62)	5.88 (4.98, 6.78)
Area under the curve	13.07 (11.93, 14.20)	14.32 (13.00, 15.64)	13.85 (12.62, 15.08)
Insulin (pmol/L)			
Fasting^b^	47.53 (35.29, 64.03)	62.84 (44.69, 88.37)	48.81 (35.28, 67.54)
2-hour^b^	255.15 (197.46, 329.70)	369.50 (275.80, 495.05)	312.99 (236.37, 414.46)
Area under the curve	617.99 (464.14, 771.83)	771.03 (594.83, 947.23)	697.47 (530.22, 864.72)
CISI (mmol/L^-1 ^* pmol/L^-1^)^b^	17.02 (13.25, 21.86)	12.38 (9.26, 16.53)	16.75 (12.78, 21.94)
Insulin_30 min_/Glucose_30 min_^b^	113.78 (80.96, 159.91)	118.24 (79.65, 175.53)	78.77 (54.70, 113.43)
HOMA-IR^b^	1.74 (1.23, 2.46)	2.33 (1.55, 3.49)	1.69 (1.16, 2.46)
Adiponectin (μg/mL)	8.90 (8.10, 9.71)^c^	8.37 (7.44, 9.30)	7.46 (6.58, 8.34)
C-Reactive protein (mg/L)^b^	1.63 (1.07, 2.47)	1.21 (0.76, 1.91)	1.24 (0.80, 1.92)
Interleukin-6 (pg/mL)^b^	1.95 (1.50, 2.54)^c^	1.67 (1.25, 2.22)	1.22 (0.93, 1.59)
Fetuin-A (μg/mL)^b^	261.52 (228.83, 298.84)	233.71 (201.54, 270.99)^c^	291.07 (212.94, 333.94)
HDL-cholesterol (mmol/L)	1.44 (1.36, 1.53)	1.39 (1.29, 1.49)	1.49 (1.40, 1.59)
LDL-cholesterol (mmol/L)	2.67 (2.46, 2.87)	2.50 (2.27, 2.73)	2.72 (2.50, 2.94)
Triglycerides (mmol/L)^b^	0.93 (0.78, 1.11)	0.99 (0.81, 1.22)	0.99 (0.81, 1.20)
			
Systolic blood pressure (mmHg)	120.57 (115.46, 125.67)	116.23 (110.39, 122.07)	119.37 (113.80 124.95)
Diastolic blood pressure (mmHg)	72.82 (69.70, 75.95)	66.69 (63.10, 70.27)^c^	72.42 (68.99, 75.84)
Heart rate (beats per minute)	67.77 (64.27, 71.26)	67.39 (63.36, 71.41)	67.63 (63.82, 71.45)

Adjusted means and associated 95% CIs were determined from analysis of covariance procedures. All models included treatment as a main effect, and baseline value and change in weight as covariates.

CI, confidence interval; CISI, composite insulin sensitivity index. HOMA, homeostasis model assessment; IR, insulin resistance; HDL, high density lipoprotein; LDL, low density lipoprotein.

^a ^Results for the Week 8 analysis were based on last-observation-carried-forward method for imputation of missing values. One observation carried forward from Week 4 visit, except fetuin-A not measured at Week 4. The Week 8 analysis was based on 41 participants, except for analysis of glucose tolerance measurements (n = 40) where one participant had a missing value for fasting glucose.

^b ^Original values were log transformed due to significant departures from normality, then back transformed to yield the geometric means and associated confidence intervals.

^c ^Significant difference compared to No Coffee (*p *< 0.05).

As compared with consuming no coffee, consumption of caffeinated coffee significantly increased adiponectin (difference in change from baseline 1.44 μg/mL; 95% CI: 0.23, 2.66) and IL-6 (60%; 95% CI: 8, 138), whereas the decrease in fetuin-A (-11%; 95% CI: -27, 9) was not statistically significant. Changes after decaffeinated coffee consumption as compared with no coffee were in the same direction and reached significance for fetuin-A (-20%; 95% CI: -35, -1), but not for adiponectin (0.91 μg/mL; 95% CI: -0.42, 2.25) and IL-6 (37%; 95% CI: -8, 103). We did not find significant differences for CRP and measures of insulin resistance or insulin secretion.

In a secondary analysis evaluating blood pressure and blood lipids (Table 3), participants using decaffeinated coffee had a reduction in blood pressure as compared with those consuming no coffee, which was statistically significant for diastolic blood pressure (systolic: -3.1 mmHg, 95% CI: -11.6, 5.3; diastolic: -5.7 mmHg, 95% CI: -10.9, -0.6). For caffeinated coffee, a small non-significant increase in blood pressure was found as compared with no coffee (systolic 1.2 mmHg, 95% CI: -6.5, 8.9; diastolic: 0.4 mmHg, 95% CI: -4.3, 5.2). There were no significant differences between treatment groups for high-or low density lipoprotein cholesterol, or fasting triglyceride concentrations.

**Figure 1:**
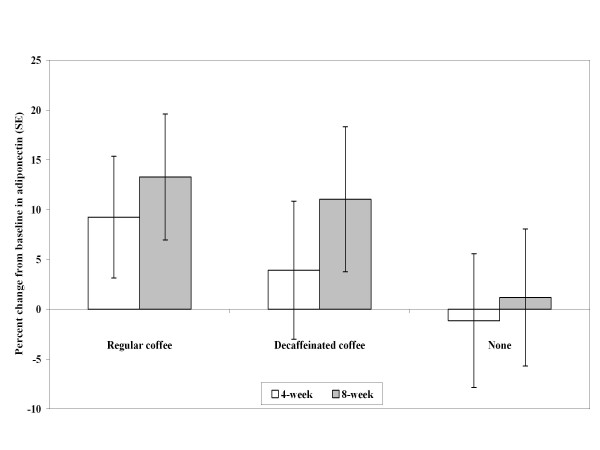
**Adjusted percentage change from baseline in adiponectin at Week 4 and Week 8 by treatment group**. Adjusted mean percent change estimates were determined from analysis of covariance models which included treatment as a main effect, and baseline value and change in weight as covariates. Error bars reflect standard errors. Regular coffee (n = 14), Decaffeinated coffee (n = 13), No coffee (n = 14).

The changes from baseline at Week 4 and Week 8 for adiponectin are shown in Figure 1. In an exploratory analysis using the complete study population regardless of treatment group, changes in adiponectin levels during the trial were significantly associated with changes in 2-hour plasma glucose (β = -0.37 mmol/L, s.e. = 0.13, p-value = 0.009) (Additional file 1, **figure s3**) and glucose AUC (β = -0.40 mmol/L, s.e. = 0.20, p-value = 0.043). Changes in IL-6 and fetuin-A were not significantly associated with changes in measures of glucose metabolism.

During the 2-week caffeine washout period prior to randomization, 15 occurrences of headaches were reported and after the baseline visit two occurrences of headaches were reported (one in the decaffeinated group and one in the control group). There were a total of four other adverse events reported during the trial that were non-serious and consisted of a urinary tract infection and gastroesophageal reflux among participants assigned to decaffeinated coffee, and a hypoglycemic event and cold sores among participants assigned to caffeinated coffee.

## Conclusions

In this 8-week randomized trial of overweight men and women, adiponectin and IL-6 levels increased for the caffeinated coffee group as compared with the group receiving no coffee. Fetuin-A levels decreased for decaffeinated coffee as compared with no coffee at the end of the intervention. There were no significant differences between the three treatment groups for CRP or measures of glucose metabolism.

Our results for adiponectin are consistent with one observational study and non-randomized trial that found higher adiponectin concentrations with increasing coffee consumption [8,10]. The effects of coffee on fetuin-A concentrations in our trial are consistent with the beneficial effects of coffee on liver function suggested by cross-sectional studies of coffee consumption and liver enzymes [9]. The lack of significant effects of coffee consumption on glucose tolerance may seem inconsistent with the effect of coffee consumption on adiponectin levels and the association between increases in adiponectin levels and improvements in glucose tolerance in our study. However, we hypothesize that the effect of coffee on glucose tolerance mediated by adiponectin levels was too modest for a study of the current sample size to detect. Possibly, long-term increases in adiponectin and reductions in fetuin-A concentrations contribute to the observed association between habitual coffee consumption and a lower risk of T2DM [1,2].

In short-term studies, caffeinated coffee intake has been associated with an acute reduction in insulin sensitivity and glucose tolerance [3,21,22]. Caffeine may reduce insulin sensitivity through adenosine receptor antagonism, or by crossing the blood-brain barrier and stimulating epinephrine release [1]. However, tolerance to these effects of caffeine may develop after longer-term intake [4]. In addition, coffee also contains a rich source of other phytochemicals such as chlorogenic acid and trigonelline, which may improve glucose metabolism through beneficial effects on oxidative stress, gluconeogenesis, gut hormones, or intestinal microflora [23]. In a randomized trial in healthy men, intake of the coffee components chlorogenic acid and trigonelline acutely reduced glucose and insulin levels 15 minutes after the oral glucose tolerance test [24]. Our findings suggest that the acute detrimental effects of caffeinated coffee on insulin sensitivity and glucose tolerance do not remain after 8 weeks of coffee consumption. In contrast to our results, four weeks of very high caffeinated coffee consumption (70 g coffee grounds) increased fasting insulin concentrations which may reflect the higher caffeine dose in that study [4].

Consistent with results from a previous trial [10], we did not find effects of coffee consumption on CRP levels. In contrast, cross-sectional studies have reported both direct or inverse associations between coffee consumption and CRP levels [8,25-27]. We found higher IL-6 levels for the caffeinated coffee group as compared with the group that drank no coffee, which is consistent with results from a cross-sectional study in Greece [25]. However, high coffee consumption did not increase IL-6 production in a previous trial in humans [10]. In our study, variation in IL-6 levels was large and baseline IL-6 levels differed between groups with low levels in the caffeinated coffee group. These limitations and the inconsistent results across studies suggest that the effects of coffee consumption on IL-6 concentrations require further study.

In a secondary analysis, caffeinated coffee consumption led to a non-significant 1.2 mmHg increase in systolic blood pressure and caffeinated and decaffeinated coffee did not affect blood lipids consistent with previous meta-analyses [28,29]. Decaffeinated coffee consumption reduced diastolic blood pressure as compared with no coffee consumption, an effect that has not been found in previous trials and requires further research [29].

Our study has several limitations that need to be considered. First, although all coffee treatment groups were blinded to the study investigators, the nature of the intervention made it impossible to completely blind the intervention assignment to the participants. Second, compliance is a concern for trials in free-living individuals. However, more than 90% of the participants completed the intervention and based on the participants' caffeine levels, compliance was high. Finally, a key limitation that may have precluded our ability to detect significant differences between the treatment groups was the modest sample size. Our results warrant the conduct of a larger long-term trial of effects of coffee on biomarkers of T2DM.

Results from cohort studies suggest that coffee consumption may reduce risk of T2DM. This study contributes to the current body of knowledge on mechanisms that may underlie this association using a randomized study design with an 8-week intervention and measurement of novel metabolic markers. Our findings suggest that improvements in adipocyte and liver function as indicated by changes in adiponectin and fetuin-A levels may contribute to beneficial metabolic effects of long-term coffee consumption. Given the popularity and widespread consumption of coffee, the effects of coffee and coffee components on metabolic risk factors warrant further investigation.

## List of abbreviations

AUC: area under the curve; BMI: body mass index; CI: confidence interval; CISI: composite insulin sensitivity index; CRP: C-reactive protein; CV: coefficient of variation; HDL: high density lipoprotein; IL-6: interleukin-6; HOMA-IR homeostasis model assessment for insulin resistance; LDL: low density lipoprotein.; OGTT: oral glucose tolerance test (OGTT); SAS: Statistical Analysis System; T2DM: type 2 diabetes mellitus; US: United States.

## Competing interests

The authors declare that they have no competing interests.

## Authors' contributions

NW contributed towards the acquisition, analysis, and interpretation of the data, drafted the manuscript, and critically reviewed the manuscript. AB contributed towards the study concept and design, the acquisition, analysis, and interpretation of the data, and critically reviewed the manuscript. QS contributed towards the acquisition, analysis, and interpretation of the data, and critically reviewed the manuscript. FH contributed towards the study concept and design, the analysis and interpretation of the data, and critically reviewed the manuscript. CM contributed towards the study concept and design, the acquisition, analysis, and interpretation of the data, and critically reviewed the manuscript. RVD contributed towards the study concept and design, the acquisition, analysis, and interpretation of the data, drafted the manuscript, critically reviewed the manuscript, and obtained funding for the study. All authors have read and approved the final manuscript.

## Supplementary Material

Additional file 1 **Additional figures and table entitled Figure S1, Figure S2, Figure S3 and Table S1**. Figure S1. Flow of participants through the study.; Figure S2. Compliance at 6-week visit determined from non-fasting serum caffeine concentrations. Shown are mean ± standard error values for caffeine and metabolites concentrations. Regular coffee (n = 14), Decaffeinated coffee (n = 13), No coffee (n = 14).; Figure S3. Association between change in adiponectin concentrations and change in 2-hour glucose concentrations during the study. Shown is a scatterplot of participant values for change from baseline at Week 8 in 2-hour glucose versus change in adiponectin including the simple linear regression line.; and Table S1. Body composition, lifestyle, and diet by coffee treatment group at baseline and the end of the trial. Click here for file
