# Supplementary material for: Effects of caffeinated and decaffeinated coffee on biological risk factors for type 2 diabetes: a randomized controlled trial
Source: Nutr J. 2011 Sep 13;10:93. doi: 10.1186/1475-2891-10-93 (PMC3180352; doi:10.1186/1475-2891-10-93)
Supplement: Additional file 1 — Additional figures and table entitled Figure S1, Figure S2, Figure S3 and Table S1. Figure S1. Flow of participants through the study.; Figure S2. Compliance at 6-week visit determined from non-fasting serum caffeine concentrations. Shown are mean ± standard error values for caffeine and metabolites concentrations. Regular coffee (n = 14), Decaffeinated coffee (n = 13), No coffee (n = 14).; Figure S3. Association between change in adiponectin concentrations and change in 2-hour glucose concentrations during the study. Shown is a scatterplot of participant values for change from baseline at Week 8 in 2-hour glucose versus change in adiponectin including the simple linear regression line.; and Table S1. Body composition, lifestyle, and diet by coffee treatment group at baseline and the end of the trial. [file 1475-2891-10-93-S1.DOC]

**Figure S1.** Flow of participants through the study

65 Assessed for Eligibility

20 Excluded

11 Not Meeting Inclusion Criteria

4 Body mass index >35 kg/m^2^

3 Hypertension

2 Body mass index <25 kg/m^2^

1 Competency

1 Abnormal lab value

9 Refused to Participate

45 Randomized

16 Randomized to Receive Caffeinated Coffee

16 Received Intervention as Assigned

14 Randomized to Receive Decaffeinated Coffee

14 Received Intervention as Assigned

15 Randomized to Receive No Coffee

15 Received Intervention as Assigned

0 Lost to Follow-up

2 Discontinued Intervention

1 Dropped out after Week 4 due to vacation

1 Dropped out after Baseline due to inconvenience

0 Lost to Follow-up

1 Discontinued Intervention

1 Dropped after Baseline due to inconvenience

0 Lost to Follow-up

1 Discontinued Intervention

1 Dropped out after Baseline due to noncompliance

15 Included in Analysis

1 Excluded from Analysis

13 Included in Analysis

1 Excluded from Analysis

14 Included in Analysis

1 Excluded from Analysis

**Figure S3.** Association between change in adiponectin concentrations and change in 2-hour glucose concentrations during the study

Shown is a scatterplot of participant values for change from baseline at Week 8 in 2-hour glucose versus change in adiponectin including the simple linear regression line.

**Table S1.** Body composition, lifestyle, and diet by coffee treatment group at baseline and the end of the trial

|  | **Caffeinated Coffee** | |  | **Decaffeinated Coffee** | |  | **No Coffee** | |
| --- | --- | --- | --- | --- | --- | --- | --- | --- |
|  | **Baseline** | **Week 8** |  | **Baseline** | **Week 8** |  | **Baseline** | **Week 8** |
|  |  |  |  |  |  |  |  |  |
| Weight (kg) | 79.8 (10.7) | 80.3 (11.7) |  | 86.5 (9.3) | 84.9 (9.7) |  | 84.6 (11.4) | 84.4 (11.3) |
| Body mass index (kg/m^2^) | 29.0 (2.3) | 29.1 (2.3) |  | 29.5 (2.2) | 29.5 (2.5) |  | 30.0 (2.4) | 30.31 (2.09) |
| Waist circumference (cm)  Men | 97.7 (7.3) | 98.7 (6.2) |  | 102.5 (7.3) | 98.1 (10.0) |  | 102.6 (6.3) | 101.2 (4.9) |
| Women | 85.2 (10.3) | 86.6 (9.1) |  | 90.9 (7.8) | 91.0 (6.6) |  | 88.3 (7.4) | 89.1 (6.3) |
| Fat mass (%)^a^ | 32.0 (7.5) | 31.9 (7.3) |  | 33.5 (8.9) | 34.4 (5.4) |  | 34.3 (6.6) | 34.5 (7.0) |
|  |  |  |  |  |  |  |  |  |
| Physical activity (MET-hrs/wk) | 28.5 (31.6) | 30.8 (33.1) |  | 37.1 (35.4) | 26.7 (17.5) |  | 31.6 (18.0) | 48.3 (44.5) |
| Daily sleep average (hours) | 6.9 (1.0) | 7.1 (1.0) |  | 6.3 (0.7) | 6.0 (2.2) |  | 7.4 (1.2) | 7.7 (1.0) |
|  |  |  |  |  |  |  |  |  |
| Total calories (kcal) | 1862.35 (388.61) | 1647.29 (441.53) |  | 1896.69 (686.46) | 1962.98 (764.02) |  | 2084.81 (551.02) | 2200.13 (788.21) |
| Protein (%) | 16.88 (3.51) | 16.99 (4.23) |  | 16.93 (2.81) | 16.28 (3.78) |  | 17.14 (5.39) | 16.68 (6.13) |
| Carbohydrate (%) | 52.50 (7.22) | 52.65 (7.82) |  | 51.46 (6.71) | 50.26 (8.63) |  | 47.98 (10.31) | 49.05 (10.93) |
| Fat (%) | 30.62 (7.63) | 31.56 (7.79) |  | 31.78 (4.57) | 33.70 (6.62) |  | 35.45 (7.29) | 29.90 (6.27) |
| Saturated fat (%) | 9.87 (3.85) | 9.76 (3.63) |  | 9.99 (2.68) | 10.52 (2.60) |  | 11.50 (4.63) | 9.18 (2.70) |
| Monounsaturated fat (%) | 10.08 (3.48) | 9.28 (3.06) |  | 9.98 (3.04) | 9.68 (3.23) |  | 11.21 (3.40) | 8.88 (3.56) |
| Polyunsaturated fat (%) | 5.72 (2.52) | 5.21 (1.78) |  | 6.66 (2.74) | 5.12 (1.38) |  | 5.46 (1.73) | 4.99 (1.81) |
| Cholesterol (mg)^b^ | 192.21  (128.45 - 326.67) | 151.54  (114.33 - 245.15) |  | 318.52  (114.20 - 387.51) | 186.52  (112.47 - 256.12) |  | 329.47  (168.31 - 486.53) | 271.83  (150.28 - 434.47) |
| Dietary fiber (g)^b^ | 16.53  (11.92 - 31.95) | 11.62  (7.52 - 19.76) |  | 14.91  (12.71 - 22.79) | 17.77  (11.75 - 40.01) |  | 17.44  (13.37 - 28.89) | 23.47  (12.42 - 39.96) |
| Total sugar (g)^b^ | 57.63  (45.49 - 98.86) | 42.28  (25.40 - 93.66) |  | 82.24  (46.92 - 130.96) | 82.26  (36.24 - 98.67) |  | 92.50  (44.46 - 108.67) | 83.63  (57.50 - 159.54) |
| Alcohol (%) | 1.14  (0.00 - 1.80) | 0.00  (0.00 - 0.37) |  | 0.00  (0.00 - 1.68) | 0.00  (0.00 - 4.01) |  | 0.02  (0.00 - 2.47) | 0.00  (0.00 - 11.81) |
| Sodium (mg)^b^ | 2537.67  (1881.92 - 3627.84) | 1732.67  (1299.60 - 2722.52) |  | 2093.36  (1479.61 - 2862.93) | 1920.78  (1296.92 – 6445.80) |  | 3184.75  (2394.19 - 4558.25) | 2476.19  (1983.45 - 5894.36) |

**Table S1.** Body composition, lifestyle, and diet by coffee treatment group at baseline and the end of the trial (continued)

|  | **Caffeinated Coffee** | |  | **Decaffeinated Coffee** | |  | **No Coffee** | |
| --- | --- | --- | --- | --- | --- | --- | --- | --- |
|  | **Baseline** | **Week 8** |  | **Baseline** | **Week 8** |  | **Baseline** | **Week 8** |
|  |  |  |  |  |  |  |  |  |
| Potassium (mg)^b^ | 1930.99  (1333.57 - 2674.08) | 1541.06  (1306.00 - 2342.55) |  | 2069.13  (1711.38 - 2296.53) | 1629.48  (1250.41 – 4311.71) |  | 2689.87  (1735.92 - 3155.19) | 2529.80  (1269.64 - 4977.11) |
| Calcium (mg)^b^ | 625.28  (549.64 - 782.08) | 744.69  (373.11 - 846.75) |  | 696.17  (547.45 - 1065.33) | 627.14  (357.19 - 2005.11) |  | 755.61  (594.55 - 1018.01) | 603.50  (377.95 - 1327.99) |
| Vitamin C (mg)^b^ | 69.01  (26.84 - 118.40) | 60.00  (34.93 - 72.05) |  | 128.41  (83.78 - 177.12) | 86.34  (36.44 - 133.54) |  | 129.81  (98.54 - 152.89) | 192.21  (81.13 - 272.06) |
| Magnesium (mg)^b^ | 213.07  (134.04 - 312.38) | 146.81  (111.61 -256.82) |  | 221.60  (184.17 - 250.46) | 170.37  (142.82 - 464.34) |  | 199.47  (176.95 - 414.46) | 215.45  (150.71 - 353.73) |
|  |  |  |  |  |  |  |  |  |

Unless otherwise noted, data are means (standard deviation) for continuous variables and number (percentages) for categorical variables.

With the exception of nutrients expressed as percent energy (%), nutrient variables are expressed as per 2000 kcal diet.

There were no significant differences at baseline examination or change from baseline over the intervention period between treatment groups for any of the above variables.

^a^ Assessed by bioelectric impedance analysis.

^b^ Due to significant departures from normality, median values and interquartile ranges are presented.
